# Effectiveness of a Web-Based Tailored Interactive Health Communication Application for Patients With Type 2 Diabetes or Chronic Low Back Pain: Randomized Controlled Trial

**DOI:** 10.2196/jmir.3904

**Published:** 2015-03-03

**Authors:** Nina Weymann, Jörg Dirmaier, Alessa von Wolff, Levente Kriston, Martin Härter

**Affiliations:** ^1^Department of Medical PsychologyCenter for Psychosocial MedicineUniversity Medical Center Hamburg-EppendorfHamburgGermany

**Keywords:** Type 2 diabetes mellitus, back pain, randomized controlled trial, health communication, Internet

## Abstract

**Background:**

The prevalence of chronic diseases such as type 2 diabetes and chronic low back pain is rising. Patient empowerment is a key strategy in the management of chronic diseases. Patient empowerment can be fostered by Web-based interactive health communication applications (IHCAs) that combine health information with decision support, social support, and/or behavioral change support. Tailoring the content and tone of IHCAs to the needs of individual patients might improve their effectiveness.

**Objective:**

The main objective was to test the effectiveness of a Web-based, tailored, fully automated IHCA for patients with type 2 diabetes or chronic low back pain against a standard website with identical content without tailoring (control condition) on patients’ knowledge and empowerment.

**Methods:**

We performed a blinded randomized trial with a parallel design. In the intervention group, the content was delivered in dialogue form, tailored to relevant patient characteristics. In the control group, the sections of the text were presented in a content tree without any tailoring. Participants were recruited online and offline and were blinded to their group assignments. Measurements were taken at baseline (t_0_), directly after the first visit (t_1_), and at 3-month follow-up (t_2_). The primary hypothesis was that the tailored IHCA would have larger effects on knowledge and patient empowerment (primary outcomes) than the control website. The secondary outcomes were decisional conflict and preparation for decision making. All measurements were conducted by online self-report questionnaires. Intention-to-treat (ITT) and available cases (AC) analyses were performed for all outcomes.

**Results:**

A total of 561 users agreed to participate in the study. Of these, 179 (31.9%) had type 2 diabetes and 382 (68.1%) had chronic low back pain. Usage was significantly higher in the tailored system (mean 51.2 minutes) than in the control system (mean 37.6 minutes; *P*<.001). Three months after system use, 52.4% of the sample was retained. There was no significant intervention effect in the ITT analysis. In the AC analysis, participants using the tailored system displayed significantly more knowledge at t_1_ (*P*=.02) and more emotional well-being (subscale of empowerment) at t_2_ (*P*=.009). The estimated mean difference between the groups was 3.9 (95% CI 0.5-7.3) points for knowledge and 25.4 (95% CI 6.3-44.5) points for emotional well-being on a 0-100 points scale.

**Conclusions:**

The primary analysis did not support the study hypothesis. However, content tailoring and interactivity may increase knowledge and reduce health-related negative effects in persons who use IHCAs. There were no main effects of the intervention on other dimensions of patient empowerment or decision-related outcomes. This might be due to our tailored IHCA being, at its core, an educational intervention offering health information in a personalized, empathic fashion that merely additionally provides decision support. Tailoring and interactivity may not make a difference with regard to these outcomes.

**Trial Registration:**

International Clinical Trials Registry: DRKS00003322; http://apps.who.int/trialsearch/Trial2.aspx?TrialID=DRKS00003322 (Archived by WebCite at http://www.webcitation.org/6WPO0lJwE).

## Introduction

Long-term conditions such as type 2 diabetes (T2D) and chronic low back pain (CLBP) are chronic diseases with high and still rising prevalence [1,2], which cause a significant burden on individuals as well as negative social and economic effects [3-8]. Thus, there is a strong need for cost-effective ways to improve the care of these long-term conditions.

To improve care of long-term conditions, patients, practitioners, scientists, and politicians have called for a greater empowerment of patients in the management of their chronic diseases [9]. Patient empowerment can be observed as a motivational construct reflecting the ability to positively influence self-management and health behavior. The main aspects of patient empowerment are knowledge of the disease, its course and treatment options, the ability to be involved in making medical decisions and relate to health care providers [10], and to manage one’s health behavior and treatment regimens [11,12]. Schulz and Nakamoto additionally stressed that these factors must be accompanied by a volitional component to better predict changes in individuals’ behavior [13]. The most popular definition of patient empowerment is probably that of Funnell et al [14] who defined patient empowerment as “the discovery and development of one’s inherent capacity to be responsible for one’s own life. People are empowered when they have sufficient knowledge to make rational decisions, sufficient control, and resources to implement their decisions, and sufficient experience to evaluate the effectiveness of their decisions”. Patient empowerment and health-related knowledge can be considered as predictors of improved self-management and health outcomes [15,16].

In times of rapidly growing Internet adoption, the Web holds the opportunity to deliver health information [17] and self-management support [18] to large numbers of participants at a comparatively low cost and at the preferred time, place, and learning speed of the individuals. Existing systematic reviews and meta-analyses of Internet interventions in somatic diseases aimed at improving lifestyles (smoking, alcohol consumption, diet, physical exercise) show promising effects on either health- or cost-related outcome measures [19]. More specifically, recent reviews and studies on Internet interventions for adults with T2D [20,21] and CLBP [22-24] also found effects on knowledge, self-efficacy, health behavioral changes, and clinical outcomes. Evidence for Internet interventions can also be found with regard to effects on more proximal outcomes such as patient empowerment [22,25,26] or specific antecedents and mediators of patient empowerment [27].

A specific application of Internet interventions combines health information with at least one other type of support, for example, social support, decision support, or behavior change support: interactive health communication applications (IHCAs). These Internet interventions are expected to improve the knowledge, involvement in decision making, motivation, and self-efficacy of users, resulting in enhanced patient empowerment [28]. This improved empowerment can then enable users to initiate changes in health behaviors, which might result in improved clinical outcomes [28,29]. A Cochrane review found that IHCAs could have positive effects on knowledge, self-efficacy, and behavioral and clinical outcomes. However, the authors demanded more evidence regarding the most suitable application and delivery approaches of IHCAs and the effects of IHCAs for different chronic diseases [28].

Still, the effectiveness of those online applications is limited by high attrition rates [30,31], and users often visit a health intervention website only once [32-34]. A major body of evidence suggests that the effect of online interventions increases with the dose (longer stays, repeated website visits, total contact hours) [35,36], and the effectiveness is maximized if patients intensively work with the information offered [37-39] and return for repeated visits [40,41].

Computer tailoring strategies such as the individualization and personalization of information, as well as an interactive presentation, have been found to effectively increase the exposure to [42] and effectiveness of Web-delivered interventions [43,44]. However, these previous studies predominantly focused on tailoring in health behavior change interventions, with great variability in how the tailoring was carried out. In addition to the question of which elements of the intervention work, one remaining challenge of research with regard to Internet interventions is finding out which delivery methods (interactivity, tailoring, individualization) are effective [45]. Therefore, evidence is especially needed with regard to disease-specific tailoring and individualization strategies in IHCAs for T2D and CLBP, focusing on more proximal outcomes such as health-related empowerment and knowledge.

In this randomized controlled trial, we compared a tailored IHCA presenting information on T2D and CLBP, self-management education, and decision support to a website presenting the same information in a content tree without tailoring. The primary hypothesis was that the tailored and individualized delivery format has a greater effect on knowledge and patient empowerment than the control website. The secondary hypothesis was that users, when facing a health decision, experience less decisional conflict and feel better prepared for the consultation after using the tailored rather than the control website. This paper reports on the trial using the two guidelines that were published in 2011 on designing and reporting Internet intervention research [18,46].

## Methods

### Study Design

We performed a blinded two-armed randomized controlled trial with a parallel design. Measurements were scheduled immediately before the first use of the system (t_1_), immediately after use (t_2_), and at 3-months follow-up (t_3_). Knowledge (primary outcome) and decisional conflict and preparation for decision making (secondary outcomes) were assessed immediately after the first visit. Patient empowerment (primary outcome) was assessed 3 months after the first visit. All measurements were online self-assessment questionnaires. The study design and procedures have been published in two study protocols [47,48]. There were no important changes to the study design, methods, or trial outcomes after trial commencement. Data collection took place between August 2012 and April 2013.

### Study Population

The eligibility criteria were age ≥18 years, access to the Internet, sufficient computer/Internet literacy, and a self-reported diagnosis of T2D or CLBP. CLBP was defined as pain in the lower back almost every day for more than 12 weeks [49].

### Recruitment

In general, based on the Cochrane review by Murray et al [28], we expected a small effect (Cohen’s *d*=0.2) of the IHCA. Based on the review by van Vugt et al [20] for diabetes, and based on a similar previous study [23] for patients with back pain, we did expect that the tailored intervention would perform better (*d*=0.2) than the control on the primary outcome knowledge for both patient groups. Based on the meta-analysis by Samoocha et al [25], we also expected a small effect with regard to the primary outcome empowerment for patients with T2D and CLBP. To detect a small effect with an alpha of .05 and a power of .80 (one-tailed *t* test), a sample size of 310 (155 per group) was required. Due to the experiences of other Internet trials [40] and the effect of incentives [50], we expected a dropout rate of 20% between registration and immediately after the first visit. Thus, we aimed to include a sample of 414 at baseline. Because we were not aware of differences in the dropout rates between T2D and CLBP patients, we calculated with the same expected dropout rate for both groups.

Recruitment took place using a number of pathways. Two pension funds and six health insurance companies were contacted to request whether they were interested in informing their insurants about the study (eg, via their website, magazine, or newsletter). Three outpatient treatment networks (in which mainly primary care and specialized practices are organized), 15 diabetology practices, 15 practices specialized in CLBP, 87 primary care practices, six rehabilitation centers and hospitals, seven patient associations, and 192 self-help groups were contacted and asked whether they were interested in displaying flyers. Additionally, information on the study and a link to it were disseminated via the mailing list of a population-representative online panel of the University of Münster. Information on the study was also available on the study website. Information and links were placed on the website of the University Medical Centre Hamburg-Eppendorf, as well as on websites that are structurally connected to the work group, one external private diabetes information website, and the website of a doctors’ and therapists’ CLBP network. An article was also published in a regional newspaper (Hamburger Abendblatt).

### Study Procedures

In this purely Web-based trial without any face-to-face component, every person meeting the eligibility criteria could register for the study on the study website (open survey on a site created exclusively for the study) by providing a unique email address and choosing a password for login. After providing online informed consent and completing the pre-assessment (T2D: eligibility criteria, demographic data, time since diagnosis, treatment; CLBP: eligibility criteria, demographic data, chronic pain grade [51]), the participants were randomly assigned to the tailored system or the control system with the content tree. The informed consent was the first page entered after login. The participants were told the approximate length of time of the survey, where data were stored and for how long, who the investigators were, and the purpose of the study. Consent was provided via checkbox. Pre-assessments were completed after providing informed consent and before randomization. Only users who had filled in the pre-assessment were allowed to use the intervention (mandatory survey). In the control condition, tailoring variables (T2D: diabetes self-care [52], barriers to insulin treatment (BIT) [53], knowledge; CLBP: coping style [54], knowledge) were assessed immediately after randomization and before the intervention. In the tailored version, coping style (CLBP) was also assessed immediately after randomization and before the intervention, whereas knowledge, diabetes self-care, and barriers to insulin treatment were assessed throughout the intervention. The reason for this is that when tailoring to coping style, the user’s coping type is determined in the beginning. At different places throughout the intervention, messages are tailored to this pre-assessed type. However, when tailoring to knowledge, diabetes self-care, and barriers to insulin treatment, there is no typology. Instead, individual items are assessed at different places throughout the intervention, and at that assessment point, one single message is tailored to the user’s answer to the single item. Immediately after their first visit to the tailored IHCA or the control website, all participants were asked to fill in the post-assessment.

All participants received an email 3 months after their first visit asking them to fill in the online follow-up questionnaire. Participants were reminded by email twice, at 2 weeks and 4 weeks after the first email. Because non-monetary incentives have been shown to reduce attrition in online trials [50,55], participants who had answered all questionnaires received a €10 Amazon gift voucher. The voucher code was sent to them by email at the end of the study.

Participants were free to use the intervention as often and as long as they wished. Between the post and follow-up assessments, no prompts or reminders were used. No recommendations were provided regarding the duration or frequency of use, but the IHCA was designed to be used in one “go”. Consequently, there were no prompts to use the intervention. No payment was required. Information on the frequency and duration of usage was gathered via server registrations. Usage data, data from the self-assessment questionnaires, and personal data such as name and email address were saved separately. Data were pseudonymized. After data collection, personal data were deleted. If participants withdrew their informed consent to study participation, their data were immediately erased. All data will be erased 5 years after the end of the study.

The study was approved by the Hamburg Medical Chamber ethics committee.

### Treatment Allocation

The informed consent outlined that participants would be randomly assigned in consecutive order (50:50) to one of two presentation formats holding the same content. The random allocation (simple randomization) of the participants was automatically performed by the software program, which also provided the website and triggered automatic emails to participants. This centralized, software-driven, computerized, simple randomization procedure to the intervention or control group assured the concealment of allocation, so that randomization could not be subverted by the team of researchers. The two formats were not further elucidated, so participants did not know whether they were in the intervention or control group.

### Description of the Intervention and Control Conditions

The tailored IHCA is designed as a stand-alone intervention that complements usual care. The T2D content of both the tailored IHCA and the control website covered basic information on diabetes (pathophysiology, epidemiology, subtypes, symptoms) and its sequelae (neuropathy, nephropathy, retinopathy, heart and vessel problems, sexual dysfunction, and depression), information on health behavior and lifestyle changes, and treatment options (see Table 1). The CLBP content covered essential information on CLBP (physiology of pain, acute vs chronic pain, chronification, epidemiology, psychological aspects, coping and pain management) and related psychological problems (depression, anxiety), diagnostic procedures, and treatment options (pharmacological and non-pharmacological; see Table 1). The look of the website (colors, font, figures, and pictures) was identical in both conditions. After registration, each participant received a password via email with which they could log onto the system as often as they wished.

**Table 1:** Overview over the IHCA contents.

Type 2 diabetes	Chronic low back pain
1. Introduction: What is this website?	1. Introduction: What is this website?
1.1. Where does the information on this site come from?	1.1. Where does the information on this site come from?
2. Basics	2. CLBP Basics
2.1. Different diabetes types	2.1. Physiological basics: back, spine, and intervertebral discs
2.2. How do I know I have type 2 diabetes?	2.2. What exactly is pain?
2.3. What causes type 2 diabetes?	2.3. What is the difference between acute and chronic pain?
2.4. How many people live with type 2 diabetes?	2.4. Why does the pain stay when the physical injury heals?
2.5. How is type 2 diabetes diagnosed?	2.5. How many people live with CLBP?
2.6. Diabetes ABCs	2.6. Managing CLBP in everyday life
2.7. Blood sugar control	3. How is CLBP diagnosed?
3. How is type 2 diabetes treated?	3.1. How much diagnostics makes sense and at which point?
3.1. What are the goals of diabetes treatment?	3.2. Diagnostic options
3.2. What can you do to treat your diabetes?	4. How is CLBP treated?
3.3. When should you consider taking pills?	4.1. How much treatment makes sense and at which point?
3.4. Insulin treatment	4.2. What is the natural, untreated course of CLBP?
3.5. Summary and overview of the treatment options	5. Are there accompanying conditions or sequelae of CLBP?
4. Acute complications and sequelae	6. Treatment options
4.1. Which acute complications can occur?	6.1. How do I recognize good treatment?
4.2. Which sequelae can occur?	7. Summary
5. Additional information and literature	8. Additional information and literature
5.1. Associations and self-help	8.1. Associations and self-help
5.2. Websites	8.2. Websites
5.3. Journals	8.3. Journals
5.4. Books	8.4. Books
6. Glossary	9. Glossary
7. Legal notice	10. Legal notice
8. References	11. References

#### Tailored Condition

In the tailored condition, the delivery format was a dialogue-based, tunneled design tailoring the content and tone of the dialogue to relevant patient characteristics. It was developed based on two preliminary studies exploring the quality of existing websites [56] and assessing patient needs [57]. A tunneled design, in which the user is guided through the content, has been found to increase website use and knowledge gained from a website more than a design with more user control [58]. Still, it might annoy the user and evoke resistance [59]. Consequently, we decided to give the user some control over the path they take through the dialogue: at the end of each text passage, the user chose one of at least three reply options. These options always included at least one answer that expressed disagreement or doubt. The user then received a tailored answer that mirrored what the user had said, respected disagreement, conveyed esteem, and empathy and built an individualized bridge to the next content block. It was not possible to skip a whole content block (meaning the subheadings in Table 1), but it was possible to view the content in more or less detail.

**Figure 1:**
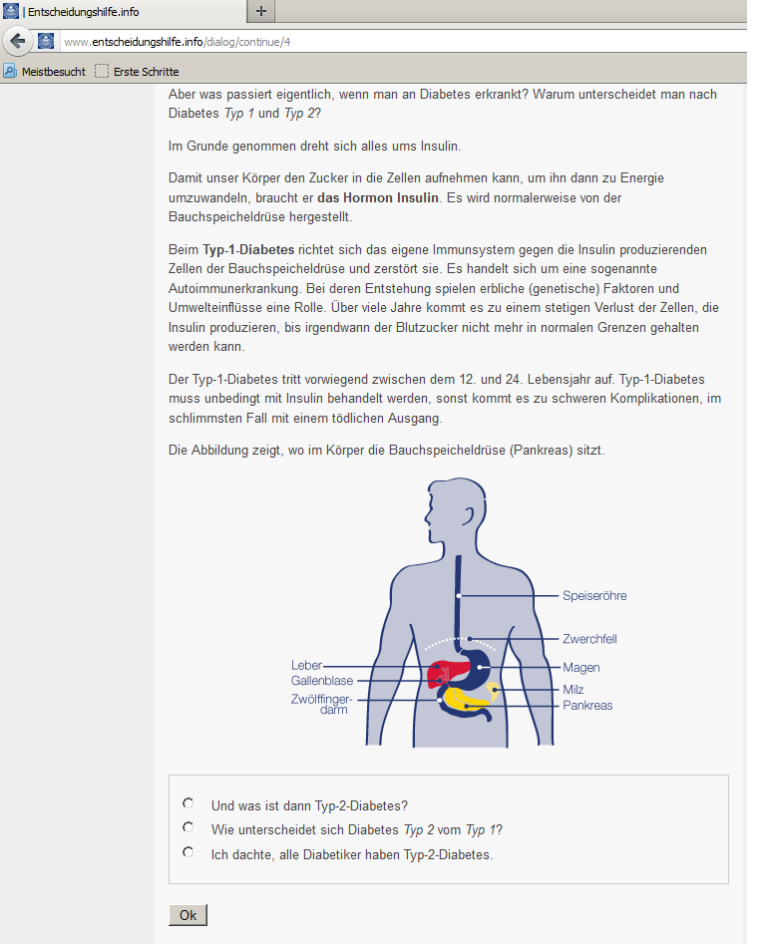
Dialogue window.

Tailoring was performed using the following characteristics for diabetes patients: current T2D knowledge and preferred level of detail, attitudes toward self-care, and, if insulin treatment was a relevant topic, psychological barriers to it. The questionnaires that assessed patient characteristics were presented during the dialogue. At the beginning of the respective section (eg, diabetic foot), the participant was asked about their knowledge or attitude toward the topic, and the following section was then modified according to their answer. Figure 1 shows such a dialogue window.

#### Diabetes Section and its Tailoring

Users’ attitudes toward self-care were assessed with items that we adapted from the Summary of Diabetes Self-care Activities Measure (SDSCA) [52] to match the respective content section (see Table 2). Users were asked how important a certain self-care activity or piece of advice is for them. Every item had three reply options: “important or very important”, “a little important”, and “not important”. The goal and techniques were inspired by Motivational Interviewing, a counseling method for addressing ambivalence about change [60].

For example, if a user attached great importance to the self-care behavior in question, this behavior was reinforced, positive consequences of the self-care behavior were stressed, and/or ideas were provided on how to keep up motivation. If a user found the self-care behavior in question “a little important”, an understanding of the users’ ambivalence was expressed, and the importance the user attached to the self-care behavior (little as it might be) was stressed and reinforced. Finally, if a user rated the self-care behavior as not important, the autonomy expressed in this answer was respected in order not to elicit resistance. Table 2 shows an example of self-care tailoring.

**Table 2:** Example of self-care tailoring: Response to “If you feel thirsty and urinate frequently, it usually means your blood sugar is…”.

Response options	Reply
High (correct answer)	That’s correct! If you want to learn more about what happens in the body and how you know that you have type 2 diabetes, you can go into more detail. Otherwise you can proceed to the next question. I’d like to learn more about that topic. I’d like to proceed to the next question.
Low (wrong answer)	No, that’s not correct. Actually, it’s the other way around: When you have type 2 diabetes, there is too much sugar in your blood. Unfortunately, you don’t realize it in the beginning. However, there are warning signs. The most important signs are […]
I don’t know	That’s ok, [name], that’s what we are here for: to learn, for example, what high blood sugar does to your body. When you have type 2 diabetes, there is too much sugar in your blood. Unfortunately, you don’t realize it in the beginning. However, there are warning signs. The most important signs are […]

Psychological barriers to insulin treatment were assessed using the Barriers to Insulin Treatment (BIT) questionnaire [53]. The BIT assesses the following expectations regarding insulin treatment: fear of injection and self-testing; expectations regarding positive insulin-related outcomes; expected hardships from insulin treatment; stigmatization by insulin injections; and fear of hypoglycemia. There are two or three items per subscale. In every item, a certain hope or fear with respect to insulin treatment is expressed (eg, “I am afraid of the pain when injecting insulin”). The user is asked to rate their agreement on a scale from 1 (completely disagree) to 10 (completely agree). A score from 1-10 can be calculated for each subscale. A validating and understanding (if a fear was expressed) or reinforcing (if a hope was expressed) answer was given. If there was a sign of fear (score >1), further information on the topic in question was provided.

#### Chronic Low Back Pain Section and its Tailoring

For CLBP, the concepts of coping style according to the avoidance endurance model (AEM) [37] and current CLBP knowledge and preferred level of detail were used for tailoring the provided information to the individual preferences of the users. The individual coping style was assessed using a questionnaire, which was presented before starting the dialogue. There are four AEM subtypes: the “depressed endurer”, which is high endurance coping (EC) and high depressiveness (D), the “happy endurer”, which is high EC and low D, the “depressed avoider”, which means low EC and high D, and the “adaptive coper”, which means low EC and low D (see Table 3). During the virtual conversation, the content, tone, and messages were tailored to the coping style of the individual user. The items that assess CLBP knowledge were presented during the dialogue. In the beginning of the respective section (eg, physiological basics), the user was asked about their level of knowledge on this subject. Depending on the response, the subsequent section was accordingly amended.

**Table 3:** Example of tailoring to coping style (CLBP).

Coping type	Adaptive coper	Happy endurer	Depressed endurer	Depressed avoider
Description of coping style	You go about your pain in a matter-of-fact manner. You know that on one hand, there is no serious disease behind it but that on the other hand, it can signal to you physical strain. You are good at taking short breaks at the right time to keep up your daily routine – maybe temporarily a little slower than usual.	You tend to keep going in your daily routine even if the pain is strong. This is, on one hand, a personal strength. However, at the same time, you run the risk of actually straining your muscles, ligaments, joints, and intervertebral discs.	You are a multi-tasker. Saying “No” to someone or not getting things done is hard on you. To meet requirements and get things done, you push yourself to your limits and beyond. Often, you don’t listen to your body before it is overstrained.	You are unsettled by your pain. You are worried that there might be a serious disease behind it, and / or you avoid activities that might increase the pain.
Take home message	Keep on like that! Make exercise part of your routine if you haven´t yet. Choose something fun and back-friendly. If you strengthen your muscles and stick to your relaxing breaks, the pain should soon vanish.	Even if it’s hard, try to pay more attention to your pain and take breaks early enough. Keep working, do things that are pleasant and fun, and keep moving – but remember to pause when you might need to!	Reconsider what you are asking from yourself: do you really have to demand so much? Maybe there are times when it is possible to leave something undone, to do it o.k. instead of perfectly, or to ask for assistance. These things are closely related to your pain.	Pain is unpleasant but not dangerous. Don’t let it suffocate you. Expand your limits step by step, and make pleasant activities a part of your everyday life.

#### Control Condition

**Figure 2:**
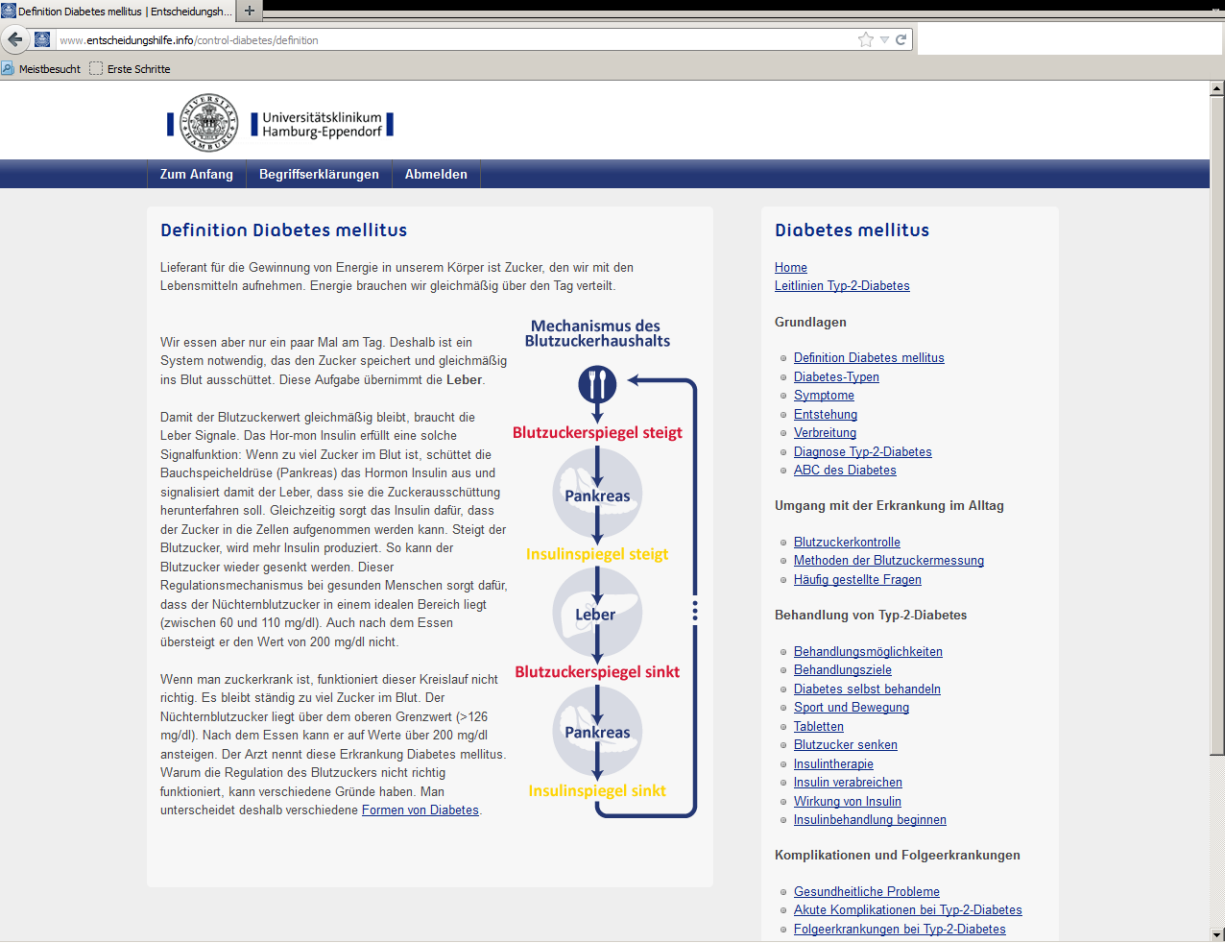
Control window.

On the control website, the content was not tailored and was not presented in a dialogue format. In contrast to the tailored, interactive version, the control website was not tunneled, and there was no guidance through the content. On the right side of each page, a content tree displayed a menu of all content sections that the participant could click on to get to the content of interest (see Figure 2). On both the intervention and control websites, the institutional affiliation of the University Medical Center Hamburg-Eppendorf was displayed at the top of each webpage.

### Potential Risks for Participants

Research focusing on the negative effects of Internet interventions is scarce. One recent study on the side effects of Internet interventions for social anxiety disorder found that 14% of participants experienced negative effects, of which the most frequent was the emergence of new symptoms [61]. Concerning long-term conditions like T2D or CLBP, possible negative effects on cognitive or emotional variables such as self-efficacy or anxiety should be considered, because of high demands concerning self-management tasks or fear-inducing information. However, several recent reviews did not find any contraindications or negative side effects of IHCAs [21,28].

### Intervention Development and Trial Design

The development process was user-oriented, evidence-based, and peer-reviewed. Two preliminary studies were conducted informing intervention development. To find out which topics are relevant to patients with T2D or CLBP, we performed a needs assessment with two steps. First, we conducted semistructured interviews with 12 physicians (T2D: 7 internists, 2 of whom were specialized in diabetology; CLBP: 5 physicians specialized in orthopedics) and 19 patients (10 with T2D, 9 with CLBP). In the second step, a self-assessment questionnaire was developed based on the main results of the interviews, and it was administered to a new and larger patient sample (T2D: N=178, CLBP: N=117). The needs assessment for T2D is described in more detail elsewhere [57]. We then conducted a cross-sectional study on the information and support available online, evaluating the formal quality, usability, and presence and quality of decision support of websites for CLBP or T2D. The results on T2D have been published elsewhere [56]. To ensure that the information is evidence-based, selected treatment guidelines were used as primary sources. Based on review articles [62,63] and up-to-dateness, the British [64] and the American [65] T2D guidelines were chosen. For CLBP, certain guidelines [49,66,67] and Cochrane reviews [68-73] were chosen. The theoretical foundations and the development of the T2D IHCA are described in more detail elsewhere [74]. Programming and graphic design were performed by the Gaia AG, a subcontractor specializing in Web-based health interventions. The intervention was not changed during the trial.

### Outcomes Assessment

The primary outcomes were knowledge (assessed immediately after the first visit) and patient empowerment (assessed at 3-months follow-up).

T2D knowledge was assessed immediately after the first visit with 16 items, and CLBP knowledge was assessed with 29 items. The items were developed to map the content covered in the sections of the tailored IHCA and could be answered with true/false/I don’t know.

For the context of long-term conditions, patient empowerment was defined as a feeling of confidence and the ability to manage the challenges resulting from the chronic disease [10]. An empowered patient can better understand and participate in care processes, use resources, and measures to reduce negative emotions, and enhance strategies to cope with chronic disease. Consequently, patient empowerment includes intrapersonal and behavioral dimensions [75]. However, at the time this study started, we could not identify a generic, adequately validated questionnaire of empowerment for general use in long-term conditions as reported by a systematic review [76]. Patient empowerment was therefore measured with the Health Education Impact Questionnaire (heiQ) [77,78]. The heiQ includes 42 items and eight dimensions: Positive and Active Engagement in Life, Health Directed Behavior, Skill and Technique Acquisition, Constructive Attitudes and Approaches, Self-Monitoring and Insight, Health Service Navigation, Social Integration and Support, and Emotional Well-being. Schuler et al [79] translated the questionnaire into German and evaluated its psychometric properties (Raykov’s Composite Reliability Coefficient, factorial and concurrent validity). They were able to replicate the structure of the eight scales and found the questionnaire to be a reliable and valid measure. We removed Social Integration and Support from our testing battery because we did not expect an effect of our IHCA on that dimension. Although these 7 heiQ scales may not comprehensively measure the multidimensional construct of empowerment given, the selected scales do cover the intrapersonal and behavioral dimensions that are part of health-related empowerment. Patient empowerment was assessed only at 3-months follow-up because we expected changes on the heiQ to take more time.

The secondary outcomes were decisional conflict and preparation for decision making, assessed immediately after the first visit. Decisional conflict was assessed with the Decisional Conflict Scale (DCS) by O’Connor [80,81]. This questionnaire measures personal perceptions of uncertainty in choosing options, modifiable factors contributing to uncertainty such as feeling uninformed, unclear about personal values, and unsupported in decision making, and effective decision making such as feeling that the choice is informed, values-based, and likely to be implemented and expressing satisfaction with the choice. Reliability is good, with a Cronbach alpha between .78 and .92 [80]. The discriminant validity is acceptable. Preparation for decision making was measured with the Preparation for Decision Making Scale (PDMS).

Preparation for decision making was measured with the Preparation for Decision Making Scale (PDMS) [82,83]. This 11-item scale assesses a patient’s or participant’s perception of how useful a decision aid or decision support intervention was in preparing them to communicate with their practitioner in making a health decision. The reliability is very good, ranging from alpha=.92 to alpha=.94. Both questionnaires were offered only to those participants who had indicated that they were facing a health decision concerning their T2D or CLBP.

To avoid missing data, all questionnaires included validation checks that alerted participants when their answers were implausible or when items were skipped. Usage data were assessed via log files. Before going online, the usability and technical functionality of the electronic questionnaire was tested by members of the research team. All outcomes were self-assessed through online questionnaires. The questionnaires were not validated for online use.

### Statistical Analysis

#### Baseline Data

Data on sample characteristics were analyzed using *t* tests (for metric data) and chi-square tests (for categorical data) to test for differences between treatment groups. A dropout analysis was performed to test for possible attrition bias. The effects of the intervention (tailored vs control condition), disease (T2D vs CLBP), gender, age, education, family status, and employment status on attrition were evaluated using *t* tests (for metric data) and chi-square tests (for categorical data).

#### Intention-to-Treat Analysis

To evaluate the effectiveness of the tailored IHCA, multiple linear regression analyses were performed using the intervention, the disease, and their interaction term as dummy-coded predictors. Intention-to-treat (ITT) and available cases (AC) analyses were performed for all outcomes. The ITT approach pooled 10 analyses, estimating missing values by a multiple regression approach using all outcomes, demographic data, and diseases but not intervention information for multiple data imputation (MI). In the primary ITT analysis, a corrected level of significance was used for testing the eight primary outcomes (Bonferroni adjustment); thus, the results with a type I error rate of *P*<.001 were considered statistically significant. For secondary outcomes, *P*<.05 was used.

#### Sensitivity Analysis (Available Cases)

The AC analysis included all of the available participants providing valid data on t_1_ and/or t_2_. In both analyses, estimated marginal means with standard errors for both the tailored and control conditions were calculated with analysis of variance (ANOVA). Additionally, these parameters were also retained for subgroups stratified by condition. In all AC analyses, results with a type I error rate of *P*<.05 were considered statistically significant. All analyses were performed using SPSS 18.0.

## Results

### Participant Flow

**Figure 3:**
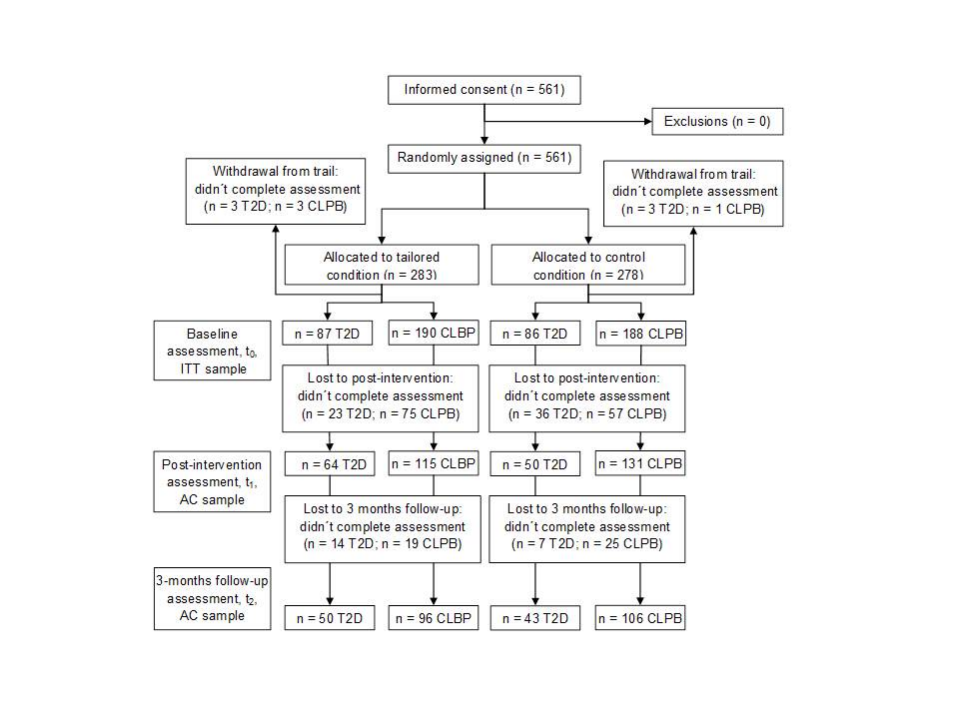
Flow of participants after randomization (ITT=intention-to-treat, AC=available cases).

A total of 561 users agreed to participate in the study. Of these, 179 (31.9%) had T2D, and 382 (68.1%) had CLBP. Analyzable data (availability of at least basic demographic information such as age and gender) at t_0_ were available from 551 users. For data analysis at t_1_, data for 360 participants was available (availability of data for at least one of the outcomes of t_1_). Three months after system use, the questionnaires of 295 participants contained data on at least one of the three outcomes at t_2_ and could thus be used for analyses (Figure 3). There were no significant differences with regard to gender, age, family status, educational level, or working status between those participants who provided all questionnaires and those who dropped out of the study after providing at least demographic data. Participants with T2D who were treated with oral anti-diabetics provided data at t_1_significantly more often than those who were treated with dietary changes or insulin. Those participants who provided data at t_1_ spent significantly more time using the system, and participants in the tailored condition spent significantly more time in the IHCA than participants in the control condition spent in the control website (see Table 4).

There was also selective dropout between t_0_ and t_1_among participants with CLBP. At t_1_, participants with CLBP were significantly (*P*=.015) younger in the tailored condition (mean 48.0; SD 12.9) than in the control condition (mean 52.0; SD 12.7). Additionally, there are significantly (*P*=.021) more participants with higher education in the tailored condition (62.6%) than in the control condition (48.9%). Among the participants with T2D, there was no selective dropout between t_0_ and t_1_. At t_2_, there were no significant differences in either of the two diseases (T2D or CLBP).

### Baseline Data

The mean age was 52.2 years (SD 13.1) in the tailored condition and 52.7 years (SD 13.0) in the control condition. Of the participants using the IHCA, 58.5% (162/277) were female (control condition: 59.1%, 162/274). There were no statistically significant differences in further demographic variables such as marital status, educational level, and working status. Sample characteristics are shown in Table 4.

**Table 4:** Sample characteristics^a^.

		Tailored condition t_0_ (n=277)	Control condition t_0_ (n=274)	Baseline differences (tailored vs control condition), *P* value	Total t_1_ (n=360)	Dropout analysis (t_1_available vs t_1_not available), *P* value
Female, n (%)		162 (58.5)	162 (59.1)	.474	216 (60.0)	.467
Age in years, mean (SD)		52.2 (13.1)	52.7 (13.0)	.668	51.8 (13.1)	.116
**Family status, n (%)**						
	Single	67 (24.2)	77 (28.1)	.742	100 (27.8)	.341
	Married	162 (58.5)	150 (54.7)		194 (53.9)	
	Divorced	39 (14.1)	37 (13.5)		52 (14.4)	
	Widowed	9 (3.2)	10 (3.6)		14 (3.9)	
Educational level, high^b^, n (%)		148 (53.4)	140 (51.1)	.322	198 (55.0)	.089
Working status, employed, n (%)		145 (55.6)	160 (58.4)	.282	207 (57.5)	.786
Years since diagnosis^c^, mean (SD)		11.1 (7.6)	10.5 (8.0)	.649	10.7 (8.2)	.858
**Current diabetes treatment** ^a^						
	Dietary change	40 (44.4)	46 (51.7)	.371	57 (50.0)	.535
	Insulin	35 (38.9)	25 (28.1)	.154	43 (37.7)	.139
	Oral anti-diabetics	59 (65.6)	55 (61.8)	.643	80 (70.2)	.023
Disability score^d^, mean (SD)		41.4 (22.5)	42.7 (22.8)	.573	42.2 (20.9)	.855
System usage in minutes, mean (SD)		51.16 (39.7)	37.6 (35.0)	<.001	49.7 (35.1)	<.001

^a^t_0_ = demographic data available (ITT population); t_1_= at least one outcome after intervention reported.

^b^more than 10 years of education.

^c^for patients with diabetes.

^d^for patients with back pain.

### Intention-to-Treat Analysis

The following results were obtained using the ITT approach including all randomized participants. The results of the sensitivity analysis using the available cases approach are reported in a separate section. Table 5 shows all of the results in detail.

#### Knowledge Immediately After the First Visit (t_1_)

With regard to knowledge of T2D or CLBP users in the tailored condition had a mean score of 77.9 (SE 1.2) compared with 76.3 (SE 1.3) in the control condition. There were no significant differences between groups (*P*=.53). There was, however, a significant difference between users with T2D and CLBP (*P*<.001), indicating higher knowledge scores in the T2D group. In addition, we observed a significant interaction effect between intervention and disease (*P*=.04), more strongly favoring the tailored condition over the control condition in CLBP (estimated mean difference of 4.6 [95% CI 1.0-8.2] points on a 0-100 points scale) than in T2D participants (estimated mean difference of -1.6 points [95% CI -7.4 to 4.2] on a 0-100 points scale).

#### Patient Empowerment at 3-Month Follow-Up (t_2_)

The heiQ does not provide a total score for patient empowerment. Table 5 shows the results for the seven included dimensions. There was no significant intervention main effect or interaction. However, there was a significant disease main effect on the dimensions Skill and Technique Acquisition (*P*=.01) and Self-Monitoring and Insight (*P*=.04), both indicating higher scores for users with T2D.

#### Decisional Conflict Immediately After the First Visit (t_1_)

There was a highly significant disease main effect. After the first use of the system, decisional conflict was lower in the CLBP group than in the T2D group (*P*<.001). There was no significant intervention main effect and no significant interaction.

#### Preparation for Decision Making Immediately After the First Visit (t_1_)

There was no significant main effect or interaction.

**Table 5:** Results of ITT and AC analyses.

			N	Tailored condition			Control condition			Intervention main effect *P*	Disease main effect *P*	Intervention x disease *P*
				T2D,M (SE)	CLBP,M (SE)	Total, M (SE)	T2D, M (SE)	CLBP, M (SE)	Total, M (SE)			
**Intention-to-treat analysis**												
	**Primary outcomes**											
		Knowledge	551	81.3 (1.9)	74.4 (1.2)	77.9 (1.2)	82.9 (2.3)	69.8 (1.4)	76.3 (1.3)	.53	<.001	.04
		Positive and active engagement in life	551	71.9 (2.5)	69.7 (1.8)	70.8 (1.4)	71.4 (2.3)	70.9 (1.8)	71.2 (1.4)	.88	.86	.43
		Health directed behavior	551	63.5 (3.9)	68.7 (2.4)	66.1 (2.4)	63.7 (3.3)	68.3 (2.4)	66.0 (2.0)	.97	.28	.92
		Emotional well-being	551	68.8 (3.9)	63.2 (2.8)	66.0 (2.6 )	62.6 (3.7)	60.2 (2.8)	61.4 (2.3)	.28	.60	.66
		Constructive attitudes and approaches	551	78.3 (2.9)	75.4 (2.1)	76.8 (1.9)	75.8 (2.5)	75.6 (1.9)	75.7 (1.6)	.498	.95	.59
		Skill and technique acquisition	551	77.6 (2.6)	65.1 (1.7)	71. 4 (1.5)	75.8 (2.9)	67.6 (1.7)	71.7 (1.8)	.62	.01	.36
		Self-monitoring and insight	551	80.1 (2.1)	70.8 (1.4)	75.4 (1.4)	79.5 (2.2)	73.4 (1.3)	76.5 (1.2)	.85	.04	.52
		Health service navigation	551	77.9 (3.1)	70.0 (2.1)	73.9 (2.0)	74.0 (2.9)	69.7 (1.8)	71.8 (1.6)	.32	.24	.44
	**Secondary outcomes**											
		Decisional conflict	551	79.7 (2.3)	61.3 (1.6)	70.5 (1.5)	75.5 (2.3)	60.3 (1.7)	67.9 (1.4)	.15	<.001	.33
		Preparation for decision making	551	60.5 (3.4)	53.8 (2.5)	56.7 (2.1)	57.6 (3.7)	51.2 (2.3)	54.4 (2.2)	.57	.14	.85
**Available cases analysis**												
	Primary outcome											
		Knowledge	330	81.1 (1.9)	77.1 (1.4)	79.1 (1.2)	81.8 (2.1)	68.7 (1.3)	75.2 (1.2)	.02	<.001	.008
		Positive and active engagement in life	295	71.8 (2.6)	69.9 (1.8)	70.9 (1.6)	71.3 (2.8)	71.3 (1.8)	71.3 (1.6)	.86	.68	.68
		Health directed behavior	295	63.0 (3.4)	69.4 (2.5)	66.2 (2.1)	64.9 (3.7)	68.7 (2.4)	66.8 (2.2)	.84	.10	.68
		Emotional well-being	295	70.8 (3.7)	66.1 (2.6)	68.5 (2.3)	60.7 (3.9)	59.3 (2.5)	60.0 (2.3)	.009	.35	.60
		Constructive attitudes and approaches	295	78.8 (2.8)	76.1 (2.0)	77.5 (1.7)	3.2 (0.09)	74.5 (3.0)	75.2 (1.9)	.30	.68	.51
		Skill and technique acquisition	295	78.3 (2.4)	64.3 (1.7)	71.3 (1.5)	75.0 (2.6)	68.8 (1.6)	71.9 (1.5)	.78	<.001	.06
		Self-monitoring and insight	295	80.3 (1.9)	70.0 (1.3)	75.2 (1.2)	79.3 (2.0)	74.7 (1.3)	77.0 (1.2)	.27	<.001	.09
		Health service navigation	295	79.1 (2.7)	71.2 (1.9)	75.2 (1.6)	73.4 (2.9)	69.8 (1.8)	71.6 (1.7)	.13	.02	.37
	**Secondary outcomes**											
		Decisional conflict	324	79.9 (2.4)	61.9 (1.8)	70.9 (1.5)	74.8 (2.7)	60.4 (1.7)	67.6 (1.6)	.13	<.001	.47
		Preparation for decision making	324	61.0 (3.3)	52.1 (2.4)	56.4 (2.0)	55.7 (3.6)	51.2 (2.2)	53.5 (2.1)	.29	.02	.47

### Sensitivity Analysis (Available Cases)

In addition to the ITT approach, we performed all calculations following the AC approach, including only participants who filled in all of the questionnaires. The aim of this procedure was to determine the extent to which missing data impacted the results reported above (sensitivity analysis).

#### Knowledge Immediately After the First Visit (t_1_)

The AC analysis showed a significant intervention main effect for knowledge (*P*=.02) indicating higher scores for the tailored condition (mean 79.1, SE 1.2) than for the control condition (mean 75.2, SE 1.2). The estimated mean difference between groups was 3.9 (95% CI 0.5-7.3) points on a 0-100 points scale. There was a significant disease x intervention interaction (*P*=.008) for knowledge, indicating the superiority of the tailored condition over the control condition in CLBP (estimated mean difference of 8.4 [95% CI 4.7-12.1] points on a 0-100 points scale) but not in T2D participants (estimated mean difference of -0.7 [95% CI -6.5 to 5.1] points on a 0-100 points scale). Additionally, there was a significant disease main effect for knowledge favoring the T2D group.

#### Patient Empowerment at 3-Month Follow-Up (t_2_)

We found a significant intervention main effect for Emotional Well-being (meaning less health-related negative effects such as anxiety, anger, and depression [78]) (*P*=.009) favoring the tailored condition (mean 68.5, SE 2.3) over the control condition (mean 60.0, SE 2.3). The estimated mean difference between groups was 25.4 (95% CI 6.3-44.5) points on a 0-100 points scale. Finally, there were significant disease main effects for Skill and Technique Acquisition (*P*<.001), Self-Monitoring and Insight (*P*<.001), and Health Service Navigation (*P*=.02) favoring the T2D group.

#### Decisional Conflict Immediately After the First Visit (t_1_)

We found a significant disease main effect (*P*<.001) showing more decisional conflict in the T2D group.

#### Preparation for Decision Making Immediately After the First Visit (t_1_)

There was a significant disease main effect (*P*=.02) indicating higher scores for the T2D compared with the CLBP group.

## Discussion

### Principal Findings

In a randomized controlled trial, we compared a Web-based, tailored, dialogue-based information system containing information on T2D or CLBP (tailored condition) with a website providing identical information without dialogue structure, tailoring, or interactive elements (control condition). The primary outcomes of the trial were knowledge and patient empowerment. Secondary outcomes were decisional conflict and preparation for decision making.

We expected that the tailored IHCA would be more attractive than the control website, be used more, and would thus lead to more knowledge and more empowerment. Indeed, participants spent significantly more time with the tailored website than the control website. Still, this did not lead to more knowledge or empowerment in the primary ITT analysis. In the AC analysis, the participants in the tailored condition displayed more knowledge at t_1_ and more Emotional Well-being at t_2_. This indicates that the tailored IHCA was more effective on these two dimensions than the control website. This was not the case for all users included; this was only the case for those who remained in the study and thus spent more time using the system. Contrary to the hypothesis, the tailored IHCA did not result in higher scores on the other six heiQ scales. It is possible that the effect was limited to the emotional level and could not be transferred to the cognitive or behavior level. This is in line with the results of Pal et al, who found that positive effects on cognitive outcomes could not be converted into behavioral changes [21]. There was a significant intervention x disease interaction favoring the tailored condition over the control condition more strongly in CLBP than in T2D participants. This superiority might indicate that tailoring in the CLBP IHCA may be more effective than tailoring in the T2D IHCA.

Other recent studies aimed directly at behavioral changes found effects on behavioral outcomes [24], and a meta-analysis on Internet-based cognitive behavioral therapy for patients with chronic somatic diseases found effects on psychological and physical outcomes [84]. A Web-based intervention aimed at psychosocial well-being in older adults with diabetes found improvements in depression, quality of life, social support, and self-efficacy [85], and a Web-based depression treatment for people with diabetes was found to reduce diabetes-specific emotional distress but had no beneficial effect on glycemic control [86]. Taken together, these results suggest that interventions aimed specifically at certain outcomes reliably have effects on these outcomes but have fewer effects on related or more distal outcomes. Consequently, our IHCA, as an educational intervention providing health information and adding behavioral change and decision support, has more consistent effects on knowledge (in persons who actually use it) than on cognitive or behavioral outcomes.

There were no significant effects regarding decisional conflict or preparation for decision making. A recent Cochrane review found that decision aids have, among other outcomes, an impact on knowledge and decisional conflict [87]. Again, the fact that we did find an impact on knowledge in the AC analysis but not on decisional conflict or preparation for decision making might be due to our IHCA being more of an educational intervention, providing the information necessary for shared decision making, than a classical decision tool.

Users with T2D yielded significantly better results regarding knowledge, preparation for decision making (only AC), and three (ITT: two) dimensions of the heiQ than participants with CLBP. One possible explanation might be that education and empowerment are traditionally cornerstones of diabetes management [88], which is not as explicitly true for the treatment of CLBP. Still, this result should be interpreted cautiously, because the instruments used to measure knowledge were different in both groups.

### Strengths and Limitations

The work presented is the first trial on a German language IHCA on T2D or CLBP. The intervention was designed carefully based on two preliminary studies. There are some limitations to the work. One limitation concerns the representativeness of the sample. Only people with Internet access could be included in the study. Of the German general population, 73% are online [89], but of the population over 50, only 47% use the Internet. Because the prevalence of both T2D [90] and CLBP increases with age [91,92], there might be a selection bias in our sample. The diagnosis was self-assessed. In addition, this presents a limitation regarding the implementation and reach of online support for these diseases. Still, attrition was comparatively low for an online trial [23]. At t_2_, 52.4% of the sample was retained. The comparatively low attrition rate in the tailored and control conditions might be due to the incentive given for complete datasets. Because none of the outcome criteria were assessed at t_0_, we cannot know whether the differences between conditions at t_1_ were caused by the intervention or had been there from the beginning.

We did not include quantitative or qualitative feedback on user acceptance. We also did not assess potential confounders (eg, which other interventions the participants used while enrolled in the study). These variables might have added to our understanding of the IHCA effects. Going beyond the scope of our study, investigating the effectiveness of the tested intervention, further research should focus on the mechanisms of change and the role of context variables through analyzing potential mediators and moderators [93]. Although the participants were blinded to the group assignment, it might be possible that participants identified the intervention group due to the unusual dialogue-based delivery format used in the intervention group. However, the design and content of both groups were nearly identical.

Another limitation arises from the measures used. First, there are concerns regarding data quality and response rates in online questionnaires [94,95]. Psychometric properties have been found to be equivalent to or even better than data obtained from paper pencil questionnaires [96,97]. There are also advantages of online assessment: data quality can additionally be improved by validation checks that alert participants if their answers are implausible or if items are skipped [96]. Furthermore, online assessment seems to be less prone to social desirability [98]. Second, only some of the measures used in this trial are standardized (DCS, PDMS, BIT), whereas others are adapted (attitudes toward self-care) for our purposes. The measure to assess the primary outcome of T2D/CLBP knowledge was developed for the purpose of this study and has not been validated. Different versions of this outcome measure with different numbers of items for T2D and CLBP are used. None of the measures have been adapted for online use, which limits their comparability to results obtained from paper pencil tests [99].

Finally, the intervention had multiple components. We cannot know which component resulted in which effect. Future research should determine which components are effective and which are not.

### Conclusions

The tailored IHCA enhanced knowledge and empowerment in persons who actually used it but failed to have effects in the total study population and on more distal outcomes. It might be concluded that tailoring and interactivity do not have effects with regard to these outcomes. Intervention components more specifically targeting cognitive and behavioral outcomes might enhance the effects. Pathways of change connecting intervention components and effects should be explored.

With regard to implementation, the IHCA could function outside of the study without major changes. Still, it would require some resources for updates and maintenance. Involving sponsors from the beginning might facilitate implementation. If our IHCA had made it to this stage, there would have been steps taken to extend its reach and effectiveness. In addition to being more specific, adaptability to tablets and mobile phones might have been an asset [21]. Another feature could be blended care to more explicitly integrate personal contacts, telephone, and online support [100]. The opportunity to share information and experiences with peers might be an especially attractive and important feature. The Pew Internet and American Life Project [101] found that people living with a chronic disease are more actively using the opportunities of Web 2.0: they generate and share content on their disease, use social media, blog, and chat more than people with no chronic conditions. Stepping into a multimedia dialogue with the users and letting expert-generated content and user-generated content spur each other might be the next step toward patient-centeredness in online support.

## Abbreviations

ADA: American Diabetes Association
AEM: avoidance endurance model
ANCOVA: analysis of covariance
DCS: Decisional Conflict Scale
heiQ: Health Education Impact Questionnaire
IHCA: Interactive Health Communication Application
PDMS: Preparation for Decision Making Scale

## Multimedia Appendix 1

CONSORT-EHEALTH checklist V1.6.2 [46].
