# Supplementary material for: Effectiveness of a Web-Based Tailored Interactive Health Communication Application for Patients With Type 2 Diabetes or Chronic Low Back Pain: Randomized Controlled Trial
Source: J Med Internet Res. 2015 Mar 3;17(3):e53. doi: 10.2196/jmir.3904 (PMC4376097; doi:10.2196/jmir.3904)
Supplement: Supplementary file 1 [file jmir_v17i3e53_app1.pdf]

CONSORT-EHEALTH Checklist V1.6.2 Report

(based on CONSORT-EHEALTH V1.6), available at [http://tinyurl.com/consort-ehealth-v1-6].

3904

Date completed

11/21/2014 9:40:13

by

Jörg Dirmaier

Effectiveness of a web-based tailored Interactive Health Communication Application for patients with type 2 diabetes or chronic low back pain: randomized controlled trial

TITLE

1a-i) Identify the mode of delivery in the title

Yes, "web-based"

1a-ii) Non-web-based components or important co-interventions in title

Yes, "tailored"

1a-iii) Primary condition or target group in the title

Yes, "for patients with type 2 diabetes or chronic low back pain"

ABSTRACT

1b-i) Key features/functionalities/components of the intervention and comparator in the METHODS section of the ABSTRACT

Yes,
"The effectiveness of the tailored IHCA was tested against a standard website (SW) with identical content without tailoring (control condition) in a blinded randomized trial with a parallel design. The content covers information on T2D and CLBP and their treatment options. In the intervention group the content was delivered in dialogue form, tailored to relevant patient characteristics. In the control group the sections of the text were presented in a content tree, without any tailoring"

1b-ii) Level of human involvement in the METHODS section of the ABSTRACT

Yes,
"The effectiveness of the tailored, fully automated IHCA was tested..."

1b-iii) Open vs. closed, web-based (self-assessment) vs. face-to-face assessments in the METHODS section of the ABSTRACT

Yes,
"Participants were recruited online and offline and blinded to group assignment. Measurements were at baseline, directly after the first visit, and at 3-month follow-up. The primary hypothesis was that the tailored IHCA has larger effects on knowledge and patient empowerment (primary outcomes) than the SWcontrol website. Secondary outcomes were decisional conflict and preparation for decision making. All measurements were online self-report questionnaires."

1b-iv) RESULTS section in abstract must contain use data

Yes,
"N = 561 users agreed to participate in the study. Of these, n = 179 (31.9%) had T2D and n = 382 (68.1%) had CLBP. System usage was significantly higher in the tailored IHCA (M = 51.2 minutes) than in the SW control group condition (M = 37.6 minutes; p < 0.001). Three months after system use, 52.4% of the sample was retained"

1b-v) CONCLUSIONS/DISCUSSION in abstract for negative trials

Yes,
"This might be due to our tailored IHCA being, at its core, an educational intervention providing health information in a personalized, empathic fashion, not a decision aid. Or tailoring and interactivity do not make a difference with regard to these outcomes. "

INTRODUCTION

2a-i) Problem and the type of system/solution

Yes,
Goals: "This trial compared a tailoredn IHCA presenting information on T2D and CLBP, self-management education and decision support in a tailored format to a website presenting the same information in a content tree without tailoring. The primary hypothesis was that the interactive and individualized delivery format has larger effects on knowledge and patient empowerment than the SWcontrol website. The secondary hypothesis was that users facing a health decision experience less decisional conflict and feel better prepared for the consultation after using the interactive and individualizedtailored site rather than the SWcontrol website. The present paper reports on the trial utilizing the two guidelines published on designing and reporting Internet intervention research in 2011 {Proudfoot, 2011, DOI: 10.1080/16506073.2011.573807;Eysenbach, 2011, 22209829}.

Problem and type of solution:
"Still, the effectiveness of those online applications is limited by high attrition rates {Murray, 2013 #90}{Habibovic, 2014 #91}{Leslie, 2005, 15530581;Glasgow, 2007, 17466816}, and oftenfew users visit a health intervention website onlymore than once {Brouwer, 2010, 19897515;Verheijden, 2007, 17478410}{Neve, 2010 #92}{Glasgow, 2011 #94}. A major body of evidence suggests that the effect of online interventions increases with dose (longer stays, repeated website visits, total contact hours) {Verheijden, 2007, 17478410;Fan, 2009, DOI: 10.1016/S1499-2671(09)31005-9}, effectiveness is maximized if patients work intensively with the information offered {Eysenbach, 2005, 15829473;Danaher, 2006, 16954125}{Donkin, 2011 #88} and return for repeated visits {Christensen, 2009, 19403466;Norman, 2007, 17888860}. Individualization and personalization of information as well as an interactive presentation have been found to effectively increase exposure to and effectiveness of interventions {Brouwer, 2011, 21212045;Boudreau, 2011, 21414998}{Kelders, 2012 #87}. These three strategies can be subsumed under the concept of tailoring {Kreuter, 2000 #64}."
"

2a-ii) Scientific background, rationale: What is known about the (type of) system

Yes,
"Trials of systematically and thoroughly developed online health interventions show small but consistent effects on behavioral and clinical outcomes {Elbert, 2014 #85}{Elbert, 2014 #85}{Samoocha, 2010, 20581001;Roshanov, 2011, 21824386;Krebs, 2010, 20558196}{Kuijpers, 2013 #86} even in older populations {Bond, 2010, 20375351}. Murray et al. {Murray, 2004, 15495094} reviewed the effects of a format that combines health information with at least one other type of support, e.g. social support, decision support, or behavior change support (= “Interactive Health Communication Applications”, IHCAs) {Murray, 2004, 15495094}. They found that IHCAs can have positive effects on knowledge, social support, clinical, and behavioral outcomes. RA recent Cochrane reviews on computer-based diabetes self-management interventions for adults with T2D found small effects on knowledge, self-efficacy, health behavior change, psychological well-being, clinical parameters, and A1c {van Vugt, 2013 #84}{Pal, 2013, 23543567}."

METHODS

3a) CONSORT: Description of trial design (such as parallel, factorial) including allocation ratio

Yes,
"This trial compared a tailoredn IHCA presenting information on T2D and CLBP, self-management education and decision support in a tailored format to a website presenting the same information in a content tree without tailoring. The primary hypothesis was that the interactive and individualized delivery format has larger effects on knowledge and patient empowerment than the SWcontrol website. The secondary hypothesis was that users facing a health decision experience less decisional conflict and feel better prepared for the consultation after using the interactive and individualizedtailored site rather than the SWcontrol website. The present paper reports on the trial utilizing the two guidelines published on designing and reporting Internet intervention research in 2011 {Proudfoot, 2011, DOI: 10.1080/16506073.2011.573807;Eysenbach, 2011, 22209829}."

3b) CONSORT: Important changes to methods after trial commencement (such as eligibility criteria), with reasons

Yes,
"There were no important changes to study design or methods after trial commencement. "

3b-i) Bug fixes, Downtimes, Content Changes

Yes
"The intervention was not changed during the trial"

4a) CONSORT: Eligibility criteria for participants

Yes,
"Eligibility criteria were age ≥18 years, access to the Internet, sufficient computer / Internet literacy and a self-reported diagnosis of T2D or CLBP. CLBP was defined as pain in the lower back almost every day for more than 12 weeks {Becker, 2004, 15614654}."

4a-i) Computer / Internet literacy

Yes,
"Eligibility criteria were age ≥18 years, access to the Internet, sufficient computer / Internet literacy and a self-reported diagnosis of T2D or CLBP. CLBP was defined as pain in the lower back almost every day for more than 12 weeks {Becker, 2004, 15614654}."

4a-ii) Open vs. closed, web-based vs. face-to-face assessments:

Yes,
Recruitment took place via a number of pathways: Two pension funds and six health insurance companies were contacted with the request whether they were interested to inform their insurants about the study (e.g. via their website, magazine, or newsletter). Three outpatient treatment networks (in which mainly primary care and specialized practices are organized), 15 diabetology practices, 15 practices specialized in CLBP, 87 primary care practices, six rehabilitation centres and hospitals, seven patient associations and 192 self-help groups were contacted and asked whether they were interested in displaying flyers. Additionally, information on the study and a link to it were disseminated via the mailing list of a population representative online panel of the University of Münster. Information on the study was also available on the study website www.entscheidungshilfe.info. Information and links were placed on the web site of the University Medical Centre Hamburg-Eppendorf as well as on websites that are structurally connected to the work group (www.psychenet.de, www.patient-als-partner.de), one external private diabetes information website (www.diabsite.de), and the website of a doctors´ and therapists´ CLBP network (http://www.agr-ev.de/en/). An article was published in a regional newspaper (Hamburger Abendblatt)."
"In this purely web-based trial without face-to-face components, Every person meeting the eligibility criteria could register for the study on the study website (open survey on a site created exclusively for the study) by providing an e-mail address and choosing a password for login."

4a-iii) Information giving during recruitment

Yes: "After providing an online informed consent (checkbox) and completing the pre-assessment (T2D: eligibility criteria, demographic data, time since diagnosis, treatment; CLBP: eligibility criteria, demographic data, chronic pain grade {Klasen, 2004, 19742049}) the participants were randomly assigned to the tailored IHCA or the SW control condition with the content tree. The informed consent was a page that was entered right after login. The participants were told the approximate length of time of the survey, which data are stored where and for how long, who the investigators are, and the purpose of the study. Consent was provided via checkbox. Pre-assessments were completed after providing informed consent and before randomization. "

4b) CONSORT: Settings and locations where the data were collected

No, not relevant. data collection was not restricted to certain settings or locations.

4b-i) Report if outcomes were (self-)assessed through online questionnaires

Yes,
"All outcomes were self-assessed through online questionnaires."

4b-ii) Report how institutional affiliations are displayed

Yes,
"Both on the intervention as well as the control website, the institutional affiliation of the University Medical Center Hamburg-Eppendorf was displayed at the top of each webpage. "

5) CONSORT: Describe the interventions for each group with sufficient details to allow replication, including how and when they were actually administered

5-i) Mention names, credential, affiliations of the developers, sponsors, and owners

Yes,
"Programming and graphic design were performed by the Gaia AG, a subcontractor specialized in web-based health interventions. "

5-ii) Describe the history/development process

Yes, in short:
"The development process was user-oriented, evidence-based and peer reviewed. Two preliminary studies were conducted informing intervention development. In order to find out which topics are relevant to patients with T2D or CLBP, we performed a needs assessment with two steps. First, semi-structured interviews with twelve physicians (T2D: seven internists, two of these specialized in diabetology; CLBP: five physicians specialized in orthopaedics) and 19 patients (ten with T2D, nine with CLBP) were conducted. In the second step, a self-assessment questionnaire was developed based on the main results of the interviews, and it was administered to a new and larger patient sample (T2D: N=178, CLBP: N=117). The needs assessment for T2D is described in more detail elsewhere {Weymann, 2014, 24916569}. We then conducted a cross-sectional study on the information and support available online, evaluating formal quality, usability, and presence and quality of decision support in websites on CLBP or T2D. The results on T2D have been published elsewhere {Weymann, 2014, 24688114}. In order to ensure that information is evidence-based, selected treatment guidelines were used as primary sources. Based on review articles {Burgers, 2002, 12401735;Stone, 2010, 19932517} and up-to-dateness, the British {National Institute for Health and Clinical Excellence (NICE), 2009 #51} and the American {American Diabetes Association (ADA), 2010, 20042772} T2D guidelines were chosen. For CLBP, guidelines {Becker, 2004, 15614654;Airaksinen, 2006, 16550448;Bundesärztekammer (BÄK), 2010 #195} and Cochrane reviews {Deshpande, 2007, 17636781;Furlan, 2008, 18843627;Hayden, 2005, 16034851;Heymans, 2004, 15494995;Staal, 2008, 18646078;Urquhart, 2008, 18253994} were chosen.

The development of the T2D IHCA is described in more detail elsewhere {Weymann, 2013, 24174871}. "

5-iii) Revisions and updating

Yes,
"The intervention was not changed during the trial."

5-iv) Quality assurance methods

Yes,
"In order to ensure that information is evidence-based, selected treatment guidelines were used as primary sources. Based on review articles {Burgers, 2002, 12401735;Stone, 2010, 19932517} and up-to-dateness, the British {National Institute for Health and Clinical Excellence (NICE), 2009 #51} and the American {American Diabetes Association (ADA), 2010, 20042772} T2D guidelines were chosen. For CLBP, guidelines {Becker, 2004, 15614654;Airaksinen, 2006, 16550448;Bundesärztekammer (BÄK), 2010 #195} and Cochrane reviews {Deshpande, 2007, 17636781;Furlan, 2008, 18843627;Hayden, 2005, 16034851;Heymans, 2004, 15494995;Staal, 2008, 18646078;Urquhart, 2008, 18253994} were chosen. "

5-v) Ensure replicability by publishing the source code, and/or providing screenshots/screen-capture video, and/or providing flowcharts of the algorithms used

Yes,
screenshots are provided.

5-vi) Digital preservation

Yes, the URL and screenshots are provided,

5-vii) Access

Yes,
"Recruitment took place via a number of pathways: Two pension funds and six health insurance companies were contacted with the request whether they were interested to inform their insurants about the study (e.g. via their website, magazine, or newsletter). Three outpatient treatment networks (in which mainly primary care and specialized practices are organized), 15 diabetology practices, 15 practices specialized in CLBP, 87 primary care practices, six rehabilitation centres and hospitals, seven patient associations and 192 self-help groups were contacted and asked whether they were interested in displaying flyers. Additionally, information on the study and a link to it were disseminated via the mailing list of a population representative online panel of the University of Münster. Information on the study was also available on the study website www.entscheidungshilfe.info. Information and links were placed on the web site of the University Medical Centre Hamburg-Eppendorf as well as on websites that are structurally connected to the work group (www.psychenet.de, www.patient-als-partner.de), one external private diabetes information website (www.diabsite.de), and the website of a doctors´ and therapists´ CLBP network (http://www.agr-ev.de/en/). An article was published in a regional newspaper (Hamburger Abendblatt)."

"Because non-monetary incentives have been shown to reduce attrition in online trials {Khadjesari, 2011, 21371988;Edwards, 2009, 19588449}, participants who had answered all questionnaires received a 10€ amazon gift voucher. The voucher code was sent to them by e-mail at the end of the study.
"

5-viii) Mode of delivery, features/functionalities/components of the intervention and comparator, and the theoretical framework

Yes,
"The tailored IHCA is designed as a stand-alone intervention complementing usual care. The T2D content of both the tailored IHCA and the control website covered basic information on diabetes (pathophysiology, epidemiology, subtypes, symptoms) and its sequelae (neuropathy, nephropathy, retinopathy, heart and vessel problems, sexual dysfunction, and depression), information on health behavior and lifestyle changes, and treatment options (see table 1). The CLBP content covered essential information on CLBP (physiology of pain, acute vs. chronic pain, chronification, epidemiology, psychological aspects, coping and pain management) and related psychological problems (depression, anxiety), diagnostic procedures, and treatment options (pharmacological and non-pharmacological, see table 1). The look of the website (colours, typing, figures and pictures) was identical in both conditions. After registration, each participant received a password via e-mail with which he/she could log onto the system as often as he/she wished. "

"In the tailored condition, the delivery format was a dialogue-based, tunnelled design tailoring the content and tone of the dialogue to relevant patient characteristics. It was developed based on two preliminary studies exploring the quality of existing websites {Weymann, 2014, 24688114} and assessing patient needs {Weymann, 2014, 24916569}. A tunnelled design where the user is guided through the content has been found to increase website use and knowledge gained from a website more than a design with more user control {Crutzen, 2012, 22532074}. Still, it might also annoy the user and evoke resistance {Danaher, 2005, 15914459}. Consequently, we decided to give the user some control over the path he/she takes through the dialogue: At the end of each text passage the user chose one of at least three reply options and received a tailored answer. The answers mirrored what the user has said, conveyed esteem and empathy and built an individualized bridge to the next content block.
Tailoring was performed using the following characteristics for diabetes patients: current T2D knowledge and preferred level of detail, attitudes towards self-care, and, if insulin treatment was a relevant topic, psychological barriers to it. The questionnaires that assessed patient characteristics were presented during the dialogue: In the beginning of the respective section (e.g. diabetic foot), the participant was asked about his knowledge or attitude toward the topic. The following section was then modified according to his/her answer. Figure 1 shows a dialogue window."

5-ix) Describe use parameters

Yes,
"Participants were free to use the intervention as often and as long as they wished, also between the post and follow-up assessment. No recommendations were provided regarding duration or frequency of use,"

5-x) Clarify the level of human involvement

Yes,
"The effectiveness of the tailored, fully automated IHCA was tested"

5-xi) Report any prompts/reminders used

Yes,
" Participants were reminded (to fill in the follow-up questionnaire) by e-mail two times, two weeks and four weeks after the first e-mail. "
However, "no prompts or reminders were used" for the use of the intervention.

5-xii) Describe any co-interventions (incl. training/support)

No co-interventions were provided.

6a) CONSORT: Completely defined pre-specified primary and secondary outcome measures, including how and when they were assessed

Yes,
"The primary outcomes were knowledge (assessed immediately after the first visit) and patient empowerment (assessed at three month follow-up). T2D knowledge was assessed with 16, CLBP knowledge with 29 items. The items were developed in order to map the content covered in the sections of the tailored IHCA, and could be answered with true / false / I don´t know. Patient empowerment was measured with the Health Education Impact Questionnaire (heiQ) {Nolte, 2007, 17027221;Osborne, 2007, 17320338}. The heiQ includes 42 items and eight dimensions: Positive and Active Engagement in Life, Health Directed Behavior, Skill and Technique Acquisition, Constructive Attitudes and Approaches, Self-Monitoring and Insight, Health Service Navigation, Social Integration and Support, and Emotional Wellbeing. Schuler and colleagues {Schuler, 2012, 22987145} translated the questionnaire into German and evaluated its psychometric properties (Raykov's Composite Reliability Coefficient, factorial and concurrent validity). They were able to replicate the structure of the eight scales and found the questionnaire to be a reliable and valid measure. We removed Social Integration and Support from our testing battery since we did not expect an effect of our IHCA on that dimension.
Secondary outcomes were decisional conflict and preparation for decision making. Decisional conflict was assessed with the Decisional Conflict Scale (DCS) by O´Connor {O'Connor, 1995, 7898294;Buchholz, 2011, ISSN: 1864-6050}. This questionnaire measures personal perceptions of uncertainty in choosing options, modifiable factors contributing to uncertainty such as feeling uninformed, unclear about personal values and unsupported in decision making, and effective decision making such as feeling the choice is informed, values-based, likely to be implemented and expressing satisfaction with the choice. Reliability is good with a Cronbach´s α between 0.78 and 0.92 {O'Connor, 1995, 7898294}. Discriminant validity is acceptable.
Preparation for decision making was measured with the Preparation for Decision Making Scale (PDMS) {Graham, 1996 #77;Buchholz, 2011, ISSN1864-6050}. This 11 item scale assesses a patient´s or participant´s perception of how useful a decision aid or decision support intervention was in preparing him or her to communicate with his or her practitioner in making a health decision. Reliability is very good ranging from α=.92 to α=.94. Both questionnaires were offered only those participants who had indicated that they were facing a health decision concerning their T2D or CLBP. In order to avoid missing data, all questionnaires included validation checks that alerted participants when their answers were implausible or items were skipped. Usage data was assessed via log files. Before going online, the usability and technical functionality of the electronic questionnaire was tested by members oft he research team. All outcomes were self-assessed through online questionnaires."

"Measurements were scheduled immediately before the first use of the system, immediately after and at three month follow-up. Knowledge (primary outcome), decisional conflict, and preparation for decision making (secondary outcomes) were assessed immediately after the first visit. Patient empowerment (primary outcome) was assessed three months after the first visit. "

6a-i) Online questionnaires: describe if they were validated for online use and apply CHERRIES items to describe how the questionnaires were designed/deployed

Yes,
"The questionnaires were not validated for online use."

6a-ii) Describe whether and how "use" (including intensity of use/dosage) was defined/measured/monitored

Yes,
"Information on frequency and duration of usage was gathered via server registrations. Usage data, data from the self-assessment questionnaires and personal data such as name and e-mail address were saved separately."

6a-iii) Describe whether, how, and when qualitative feedback from participants was obtained

Yes,
"We did not include quantitative or qualitative feedback on user acceptance"

6b) CONSORT: Any changes to trial outcomes after the trial commenced, with reasons

Yes,
"There were no important changes to study design, methods or trial outcomes after trial commencement"

7a) CONSORT: How sample size was determined

7a-i) Describe whether and how expected attrition was taken into account when calculating the sample size

Yes,
"Based on the Cochrane review by Murray et al. {Murray, 2004, 15495094} we expected a small effect on the primary outcomes (Cohen's d=0.2). To detect a small effect with an α of 0.05 and a power of 0.80 (one-tailed t-test), a sample size of N=310 (155 per group) was required. Expecting a dropout rate of 20% between registration and follow-up (3 months), we aimed at including a sample of N=414 at baseline."

7b) CONSORT: When applicable, explanation of any interim analyses and stopping guidelines

Not applicable

8a) CONSORT: Method used to generate the random allocation sequence

Yes,
"The informed consent informed participants that they would be randomly assigned to one of two presentation formats holding the same content. The two formats were not further elucidated so participants did not know whether they were in the intervention or control group. Randomization was performed automatically by the software providing the website, programmed by the Gaia AG"

8b) CONSORT: Type of randomisation; details of any restriction (such as blocking and block size)

Not applicable, see above

9) CONSORT: Mechanism used to implement the random allocation sequence (such as sequentially numbered containers), describing any steps taken to conceal the sequence until interventions were assigned

Not applicablem see above

10) CONSORT: Who generated the random allocation sequence, who enrolled participants, and who assigned participants to interventions

Yes,
"Randomization was performed automatically by the software providing the website, programmed by the Gaia AG."

11a) CONSORT: Blinding - If done, who was blinded after assignment to interventions (for example, participants, care providers, those assessing outcomes) and how

11a-i) Specify who was blinded, and who wasn't

Yes,
"The two formats were not further elucidated so participants did not know whether they were in the intervention or control group."

11a-ii) Discuss e.g., whether participants knew which intervention was the "intervention of interest" and which one was the "comparator"

Yes,
"Although the participants were blinded to group assignment, it might be possible that participants knew which was the intervention group due to the unusual dialogue-based delivery format used in the intervention group. However, design and content of both groups was identical. "

11b) CONSORT: If relevant, description of the similarity of interventions

Yes,
"On the control website, the content was not tailored and not presented in a dialogue format. In contrast to the tailored, interactive version, it was not tunnelled; there was no guidance through the content. On the right of each page a content tree displayed a menu of all content sections that the participant can click on to get to the content of interest (see figure 2). Both on the intervention as well as the control website, the institutional affiliation of the University Medical Center Hamburg-Eppendorf was displayed at the top of each webpage. "

12a) CONSORT: Statistical methods used to compare groups for primary and secondary outcomes

Yes,
"Baseline data
Data on sample characteristics were analysed using t-tests (for metric data) and χ2-tests (for categorical data) to test for differences between treatment groups. A drop-out analysis was performed to test for possible attrition bias. The effect of intervention (tailored vs. control condition), condition (T2D vs. CLBP), gender, age, education, family status, and employment status on attrition was evaluated using t-tests (for metric data) and χ2-tests (for categorical data).
Intention-to-treat analysis
To evaluate the effectiveness of the tailored IHCA, multiple linear regression analyses were performed using intervention, condition, and their interaction term as dummy-coded predictors.
An intention-to-treat analysis (ITT) and an available cases analysis (AC) were performed for all outcomes. The ITT approach pooled ten analyses estimating missing values by a multiple regression approach using all outcomes, demographic data and conditions but not intervention information for multiple data imputation (MI). In the primary ITT analyses, a corrected level of significance was used for testing the eight primary outcomes (Bonferroni adjustment), thus, results with a type I error rate of $p < 0.00625$ were considered statistically significant. For secondary outcomes, $p < 0.05$ was used.
Sensitivity analysis (available cases)
The AC analysis included all the available participants providing valid data on t1 and/or t2. In both analyses, estimated marginal means with standard errors for both the tailored and control conditions were calculated with analysis of variance (ANOVA). Additionally, these parameters were also retained for subgroups stratified by condition. In all AC analyses, results with a type I error rate of $p<0.05$ were considered statistically significant. All analyses were performed using the SPSS 18.0 (Chicago, IL)."

12a-i) Imputation techniques to deal with attrition / missing values

Yes,
"The ITT approach pooled ten analyses estimating missing values by a multiple regression approach using all outcomes, demographic data and conditions but not intervention information for multiple data imputation (MI)."

12b) CONSORT: Methods for additional analyses, such as subgroup analyses and adjusted analyses

Yes,
"Sensitivity analysis (available cases)
The AC analysis included all the available participants providing valid data on t1 and/or t2. In both analyses, estimated marginal means with standard errors for both the tailored and control conditions were calculated with analysis of variance (ANOVA). Additionally, these parameters were also retained for subgroups stratified by condition. In all AC analyses, results with a type I error rate of $p<0.05$ were considered statistically significant. All analyses were performed using the SPSS 18.0 (Chicago, IL)."

RESULTS

13a) CONSORT: For each group, the numbers of participants who were randomly assigned, received intended treatment, and were analysed for the primary outcome

Yes,
see text, tables and figures in text (cannot copy it here).

13b) CONSORT: For each group, losses and exclusions after randomisation, together with reasons

Yes,
see Figure 3.

13b-i) Attrition diagram

No, we we were not able to analyze this because this data was not provided by the fully automated software providing the website and the data collection tool.

14a) CONSORT: Dates defining the periods of recruitment and follow-up

Yes,
"Data collection took place between and August 2012 and April 2013"

14a-i) Indicate if critical "secular events" fell into the study period

No, not relevant.

14b) CONSORT: Why the trial ended or was stopped (early)

not applicable, trial did not end early.

15) CONSORT: A table showing baseline demographic and clinical characteristics for each group

Yes,
see Table 4.

15-i) Report demographics associated with digital divide issues

Yes,
see Table 4

16a) CONSORT: For each group, number of participants (denominator) included in each analysis and whether the analysis was by original assigned groups

16-i) Report multiple "denominators" and provide definitions

No
We did not report this analyses because as mentioned above, the system did not provide this kind of data.

16-ii) Primary analysis should be intent-to-treat

Yes.
"The following results were obtained using the ITT approach including all randomized participants. The results of the sensitivity analysis using the PP approach are reported in a separate section. "

17a) CONSORT: For each primary and secondary outcome, results for each group, and the estimated effect size and its precision (such as 95% confidence interval)

Yes,
e.g. "The estimated mean difference between groups was 3.9 [95%CI: 0.5-7.3] points on a 0 to 100 points scale"

17a-i) Presentation of process outcomes such as metrics of use and intensity of use

Yes,
"
System usage in minutes
M (SD)
51.16 (39.7)
37.6 (35.0)
<0.001
49.7 (35.1)
<0.001
"

17b) CONSORT: For binary outcomes, presentation of both absolute and relative effect sizes is recommended

Not applicable

18) CONSORT: Results of any other analyses performed, including subgroup analyses and adjusted analyses, distinguishing pre-specified from exploratory

Yes,
"In addition to the ITT approach we performed all calculations following the AC approach including only participants that have filled in all the questionnaires. "

18-i) Subgroup analysis of comparing only users

Yes,
"Intention-to-treat analysis (ITT) and available cases analysis (AC) were performed for all outcomes."

19) CONSORT: All important harms or unintended effects in each group

Not applicable

19-i) Include privacy breaches, technical problems

Not applicable, not available

19-ii) Include qualitative feedback from participants or observations from staff/researchers

Not available

DISCUSSION

20) CONSORT: Trial limitations, addressing sources of potential bias, imprecision, multiplicity of analyses

20-i) Typical limitations in ehealth trials

Yes,
"Strengths and limitations
The work presented is the first trial on a German language IHCA on T2D or CLBP. The intervention was designed carefully based on two preliminary studies.
There are some limitations to the work presented. One limitation is representativeness of the sample: Only people with Internet access could be included in the study. 73% of the German general population are online {van Eimeren, 2011 #28}, but of the population over 50, only 47% use the Internet. Since the prevalence of both T2D {Centers for Disease Control and Prevention (CDC), 2012 #127} and CLBP increases with age {Göbel, 2001, 11810338;Gesundheitsberichterstattung des Bundes (GBE), #203} there might be a selection bias in our sample. The diagnosis was self-assessed. In addition, this presents a limitation regarding implementation and reach of online support for these conditions. Still, attrition was comparatively low for an online trial {Simon, 2012, 22154867}. At t2, 52.4% of the sample were retained. The comparatively low attrition rate in the tailored and in the control condition might be due to the incentive given for complete datasets. Since none of the outcome criteria were assessed at t0, we cannot know whether differences between conditions at t1 are caused by the intervention or if they had been there from the beginning.
We did not include quantitative or qualitative feedback on user acceptance. Neither did we assess potential confounders (e.g., which other interventions the participants used while being in the study). These variables might have added to our understanding of the IHCAs effects. Although the participants were blinded to group assignment, it might be possible that participants knew which was the intervention group due to the unusual dialogue-based delivery format used in the intervention group. However, design and content of both groups was nearly identical.
Another limitation arises from the measures used. Firstly, there are concerns regarding data quality and response rates in online questionnaires {Wyatt, 2000, 10887170;Best, 2001, DOI: 10.1177/089443930101900201}. Psychometric properties have been found to be equivalent with data obtained from paper pencil questionnaires or even better {van Gelder, 2010, 20880962;Pouwer, 1998, 9481149}. There are even advantages of online assessment: Data quality can additionally be improved by validation checks that alert participants when their answers are implausible or items are skipped {van Gelder, 2010, 20880962}. Furthermore, online assessment seems to be less prone to social desirability {Booth-Kewley, 2007, DOI: 10.1016/j.chb.2004.10.020}. Secondly, only some of the measures used in the present trial are standardized (DCS, PDMS, BIT) while others are adapted (attitudes toward self-care) for our purposes. The measure to assess the primary outcome of doiabetes / CLBP knowledge was developed for the purpose of this study and has not been validated. Different versions of this outcome measure, with different numbers of items for T2D and CLBP, are used. None of the measures have been adapted for online use which limits their comparability to results obtained from paper pencil tests {Buchanan, 2003, 16291542}.
Finally, the intervention had multiple components. We cannot know which component resulted in which effect. Future research should determine which components are effective and which are not."

21) CONSORT: Generalisability (external validity, applicability) of the trial findings

21-i) Generalizability to other populations

Yes,
"One limitation is representativeness of the sample: Only people with Internet access could be included in the study. 73% of the German general population are online {van Eimeren, 2011 #28}, but of the population over 50, only 47% use the Internet. Since the prevalence of both T2D {Centers for Disease Control and Prevention (CDC), 2012 #127} and CLBP increases with age {Göbel, 2001, 11810338;Gesundheitsberichterstattung des Bundes (GBE), #203} there might be a selection bias in our sample. The diagnosis was self-assessed. In addition, this presents a limitation regarding implementation and reach of online support for these conditions. Still, attrition was comparatively low for an online trial {Simon, 2012, 22154867}. At t2, 52.4% of the sample were retained. The comparatively low attrition rate in the tailored and in the control condition might be due to the incentive given for complete datasets. Since none of the outcome criteria were assessed at t0, we cannot know whether differences between conditions at t1 are caused by the intervention or if they had been there from the beginning."

21-ii) Discuss if there were elements in the RCT that would be different in a routine application setting

Not applicable

22) CONSORT: Interpretation consistent with results, balancing benefits and harms, and considering other relevant evidence

22-i) Restate study questions and summarize the answers suggested by the data, starting with primary outcomes and process outcomes (use)

Yes,
"In a RCT we compared a web-based, tailored, dialogue-based information system that contains information on T2D or CLBP (tailored condition) to a website providing identical information without dialogue structure, tailoring or interactive elements (control condition). The primary outcomes of the trial were knowledge and patient empowerment. Secondary outcomes were decisional conflict and preparation for decision making. "

22-ii) Highlight unanswered new questions, suggest future research

Yes,
"With regard to implementation, the IHCA could function outside the study without major changes. Still, it would need some resources for updates and maintenance. Involving sponsors from the beginning might facilitate implementation. If our IHCA had made it to this stage, there would have been steps to take in order to extend its reach and effectiveness. Besides being more specific, adaptability to tablets and mobile phones might have been an asset {Pal, 2013, 23543567}. Another could be blended care, more explicitly integrating personal contacts, telephone, and online support {Brouwer, 2011, 21212045}. The opportunity to share information and experiences with peers might be an especially attractive and important feature. The Pew Internet and American Life Project {Fox, 2010 #17} found that people living with a chronic condition are more actively using the opportunities of web 2.0: they generate and share content on their condition, use social media, blog, and chat more than people with no chronic condition. Stepping into a multimedia dialogue with the users and letting expert-generated content and user-generated content spur each other might be the next step towards patient-centeredness in online support.
"

Other information

23) CONSORT: Registration number and name of trial registry

Yes
"Trial registration: International Clinical Trials Registry DRKS00003322"

24) CONSORT: Where the full trial protocol can be accessed, if available

Yes,
"Study design and procedures have been published in two study protocols {Dirmaier, 2013, 23768119;Weymann, 2013, 23406466}"

25) CONSORT: Sources of funding and other support (such as supply of drugs), role of funders

Yes
"This study was funded by the German Federal Ministry of Education and Research (grant number: 01GX0710)."

X26-i) Comment on ethics committee approval

Ves,
"The study was approved by the Hamburg Medical Chamber ethics committee."

x26-ii) Outline informed consent procedures

Yes,
"The informed consent was a page that was entered right after login. The participants were told the approximate length of time of the survey, which data are stored where and for how long, who the investigators are, and the purpose of the study. Consent was provided via checkbox. "

X26-iii) Safety and security procedures

Yes,
"Usage data, data from the self-assessment questionnaires and personal data such as name and e-mail address were saved separately. Data were pseudonymized. After data collection, personal data were deleted. If a participant withdrew his or her informed consent to study participation his or her data were erased immediately. All data will be erased five years after the end of the study. "

X27-i) State the relation of the study team towards the system being evaluated

Yes,
"NW, JD, and MH were among the developers of the intervention. All authors declare that they have no competing interests."
